# Changes in prevalence and socioeconomic inequalities in secondhand smoke exposure in Spanish children, 2016–2019

**DOI:** 10.18332/tid/189392

**Published:** 2024-06-21

**Authors:** Marta Sanz-Mas, Xavier Continente, Elisabet Henderson, Esteve Fernández, Anna Schiaffino, Mónica Pérez-Ríos, Albert Espelt, Mònica Guxens, Maria José López

**Affiliations:** 1 Servei d’Avaluacio i Metodes d’Intervencio (SAMI), Agencia de Salut Publica de Barcelona (ASPB), Barcelona, Espana; 2 Departament de Medicina i Ciencies de la Vida (MELIS), Universitat Pompeu Fabra, Barcelona, Espana; 3 Centro de Investigacion Biomedica en Red de Epidemiologia y Salud Publica (CIBERESP), Madrid, Espana; 4 Institut de Recerca Sant Pau (IR SANT PAU), Barcelona, Espana; 5 Institut d’Investigacio Biomedica de Bellvitge (IDIBELL), Institut Catala d’Oncologia (ICO), L’Hospitalet de Llobregat, Barcelona, Espana; 6 Facultat de Medicina, Universitat de Barcelona, Barcelona, Espana; 7 Centro de Investigacion Biomedica en Red de Enfermedades Respiratorias (CIBERES), Madrid, Espana; 8 Departament de Salut, Direccio General de Planificacio en Salut, Generalitat de Catalunya, Barcelona, Espana; 9 Area de Medicina Preventiva y Salud Publica, Universidad de Santiago de Compostela, Santiago de Compostela, Espana; 10 Departament de Psicobiologia i Metodologia de les Ciencies de la Salut, Universiat Autonoma de Barcelona, Barcelona, Espana; 11 Instituto de Salud Global de Barcelona (ISGlobal), Barcelona, Espana; 12 Department of Child and Adolescent Psychiatry/Psychology, Erasmus MC, University Medical Centre, Rotterdam, The Netherlands

**Keywords:** tobacco smoke pollution, smoke-free rules, children, inequalities, socioeconomic position

## Abstract

**INTRODUCTION:**

Children are vulnerable to secondhand smoke (SHS) exposure, especially those with lower socioeconomic status. This study assesses the changes in prevalence and socioeconomic inequalities in SHS exposure in children younger than 12 years old in Spain between 2016 and 2019.

**METHODS:**

We conducted two cross-sectional studies among representative samples of households with children aged <12 years in Spain, in 2016 (n=2411) and 2019 (n=2412). Families were interviewed to assess children’s SHS exposure in private settings and outdoor public venues and their adoption of home and car smoke-free rules. We used the education level of the home main earner as a proxy for socioeconomic position. Changes over time in the prevalence and socioeconomic inequalities of SHS exposure and smoke-free rules were estimated through adjusted Poisson regression models with robust variance according to sociodemographic covariates (adjusted prevalence ratios, APRs).

**RESULTS:**

In 2019, 70.5% of children were exposed to SHS in Spain. No changes between 2016 and 2019 were found for overall SHS exposure, exposure at home, and at school entrances. SHS exposure increased at public transport stations (APR=1.24; 95% CI: 1.03–1.49) and outdoor hospitality venues (APR=1.17; 95% CI: 1.07–1.29) while it decreased in cars (APR=0.74; 95% CI: 0.56–0.98) and parks (APR=0.87; 95% CI: 0.77–0.98). Households with lower education level had higher prevalence of SHS exposure at home in 2019 compared with those with university studies (primary: APR=1.30; 95% CI: 1.11–1.51; secondary: APR=1.12; 95% CI: 1.00–1.25) and were less likely to adopt home indoor smoke-free rules (primary: APR=0.88; 95% CI: 0.79–0.99; secondary: APR=0.95; 95% CI: 0.89–1.02). Socioeconomic inequalities in SHS exposure at home persisted between 2016 and 2019 (p>0.05), while decreased in smoke-free rules in cars (p=0.039).

**CONCLUSIONS:**

Reported SHS exposure among children in Spain remained high between 2016 and 2019. Inequalities persisted at home, highlighting the need for measures to reduce such exposure with an equity perspective.

## INTRODUCTION

Secondhand smoke (SHS) causes thousands of deaths and disabilities every year worldwide^1,2^. Children are particularly vulnerable to its health effects and depend on the adult’s choice for smoke-free environments^3^. SHS exposure increases the risk for sudden infant death syndrome, low birth weight, lower respiratory infections, middle ear disease, and more severe episodes of asthma in children^4,5^. In Spain, 136403 incident cases of respiratory diseases in children were attributable to SHS exposure at home in 2015^6^. Furthermore, children exposed to SHS become more susceptible to smoking initiation later in life^7^.

In Spain, Law 42/2010^8^ banned smoking in 2011 in all enclosed public places and workplaces, and recognized children as a vulnerable population to the effects of tobacco use, leading to specific measures, like the smoking prohibition in all school premises and playgrounds. Still, smoke-free policies in Spain do not cover other outdoor and private settings, such as households or cars, where children might spend a great amount of time. SHS exposure prevalence among Spanish children was assessed at the national level in 2016, showing that about three out of four children aged <12 years were exposed to SHS^9^.

SHS exposure at early ages and adoption of smoke-free rules at private settings has been widely associated with socioeconomic position (SEP). Children from families with a disadvantaged SEP are more likely to be exposed to SHS and less likely to live in households with smoke-free rules^10-12^. In Spain, some studies have reported a higher risk of SHS exposure at private settings among children whose parents had a lower education level and families with more deprived social class^9,13^.

In different European countries, children’s SHS exposure at home has declined over the years across all socioeconomic groups, but inequalities in exposure persisted^10^ or increased^12^. So far, only one study in Spain carried out in 2016 has examined children’s SHS exposure prevalence at the national level in different settings, and its association with the SEP^9^. Therefore, our study aims to assess the changes in the prevalence and potential socioeconomic inequalities in SHS exposure at different private and outdoor public venues among children aged <12 years as well as the adoption of home and car smoke-free rules in Spain between 2016 and 2019.

## METHODS

### Study design, setting and participants

We conducted two cross-sectional studies based on two telephone surveys among representative samples of households with children aged <12 years living in Spain in November 2016 and November 2019. The same methodology was followed in both surveys.

In brief, 2411 households with children aged <12 years in Spain were selected in 2016 proportionally according to different sociodemographic characteristics (geographical region, size of municipality of residence, and child’s sex and age) to ensure representativity. Households were contacted through randomly selected landlines and mobile phones to limit a potential selection bias^14^. Households were randomly selected from a landline telephone directory proportionally according to the previously established strata. Likewise, mobile phone numbers were randomly generated from existing prefixes and people with mobile phones who did not have a landline in the household were included in the study, representing almost 30% of the sample. Non-residential landlines were excluded. Only households with at least one child younger than 12 years old were included in the study, according to previously established quotas (child’s sex and age). For the 2019 survey, we invited former 2016 survey participating households with children who remained within the age limit of 12 years. A total of 23.3% (n=562) of homes that had participated in 2016 were interviewed again in 2019. Non-responses and rejections were replaced with other households from the same quotas established in 2016 regarding the sociodemographic characteristics. New households were also contacted to include families with children younger than 3 years old in 2019 to keep sample representativeness in terms of the age quota. The final sample of the 2019 survey comprised 2412 households with children aged <12 years .

### Measures and variables

We administered questionnaires via computer-assisted telephone interviews to parents or legal guardians. Both questionnaires (2016 and 2019) were designed based on another questionnaire used in previous studies to estimate adult’s SHS exposure^15,16^ adapted for children for the 2016 survey^9^.

*SHS exposure and voluntary smoke-free rules*

Parents or legal guardians were asked about children’s SHS exposure in private settings (home and car), and different outdoor public settings, including public transport stations, school and nursery outdoor gates, outdoor areas of hospitality venues, parks, and children’s playgrounds. Information on smoke-free rules adopted in homes and cars was also collected.

Children were considered exposed to SHS at home when living with at least one household member regularly smoking inside the home or its outdoor areas (balconies/terraces). Indoor and outdoor exposure was also assessed separately. Car exposure was categorized based on the time the child spent in a private car while someone smoked in the previous week. Only children whose parents reported to be exposed 0 min/day were defined as ‘unexposed’.

SHS exposure at public transport stations was assessed by asking if the child had used a means of public transport in which someone had smoked at the transport stop in the previous week (yes/no). For school or nursery outdoor gates, children were considered ‘exposed’ if someone had smoked at the entrance/exit door in the child’s presence in the previous week (yes/no; does not go to school or nursery). Regarding outdoor areas of hospitality venues, parks, and children’s playgrounds, children were considered exposed if parents reported their child had been exposed to SHS in the previous week at that place (yes/no). All settings were assessed in 2016 and 2019, except children’s playgrounds, assessed only in the 2019 survey.

Overall exposure in private settings was defined as being exposed at home and/or in the car. Overall exposure in outdoor public settings was defined as being exposed in at least one of the outdoor public settings studied. Overall exposure to SHS was defined as being exposed in at least one of the settings studied. SHS exposure at children’s playgrounds was not included in the overall variables to be able to compare between both survey years.

Voluntary smoke-free rules in homes were examined with the following questions: ‘What is the situation that better describes the rules about smoking inside your home?’ (smoking is not allowed anywhere/only allowed in some rooms/ only allowed on special occasions/ allowed anywhere), and ‘Is smoking allowed in the outdoor areas of the household such as in the terrace, gallery, balcony or garden?’ (yes/no). Based on that, we created a variable with the following categories: ‘Only indoor bans’, ‘Indoor and outdoor bans’ and ‘No bans’. We determined car smoke-free rules based on the question: ‘Is smoking allowed in the car (or one of the cars if there is more than one)?’ (yes/no).

*Socioeconomic position (SEP)*

To assess socioeconomic inequalities in the study outcomes, we used the education level attainment of the main earner of the household as a proxy indicator of the household’s SEP^17^, defined into three categories: 1) ‘primary studies or lower’ including people without studies who can or cannot read and/or write, and people with up to 8 years of schooling; 2) ‘secondary studies’ including people with up to 12 years of schooling, vocational training and/or baccalaureate; and 3) ‘university studies’ including people with Bachelor’s degree, Master’s degree, and/or doctorate.

*Covariates*

Sociodemographic variables were included to describe the study sample and control for potential confounders. We considered child’s sex, age, and country of birth. We also took into account the family relationship (mother/father/others), age, country of birth, and smoking status (smoker or non-smoker) of the respondent.

### Ethical approval

The study protocol was approved by the Comité de Ética de Investigación Clínica del Parc de Salut Mar (code 2017/7388/I). All procedures were performed in compliance with national and international guidelines (deontological code, Declaration of Helsinki) and the Organic Law 15/1999 of December 13 on the Protection of Personal data. Participants were informed about the study’s objective and their right to withdraw.

### Data analysis

A descriptive analysis of all sociodemographic variables was performed for 2016 and 2019. A comparison between years was conducted using chi-squared test. We built Poisson regression models with robust variance to assess changes in SHS exposure and smoke-free rules between 2016 and 2019. First, overall and site-specific SHS exposure prevalences and the prevalences of families adopting smoke-free rules in private settings were calculated for 2016 and 2019 with their corresponding 95% confidence intervals (CI), using margins when the other independent variables had their values at their means. Then, we estimated prevalence ratios (PR) and the corresponding 95% CI, as recommended for variables with high prevalence^18^. PR is defined as ‘the prevalence in exposed population divided by the prevalence in non-exposed’^19^ (in this case, 2019 vs 2016). This metric estimates the association between the dependent variable (SHS exposure or smoke-free rules) and independent variable (survey year). To take into account potential influence of other variables that might affect the outcome, we also estimated the adjusted prevalence ratio (APR) by building multivariate models adjusted by child’s sex and age, family relationship, age, and smoking status of the respondent, and education level of the home main earner. Finally, we built multivariate Poisson regression models with robust variance adjusted by abovementioned confounding variables and including an interaction term between year and education level of the home main earner. APRs were estimated by education level of the main earner of the household for 2016 and 2019 (university studies as the reference category). The interaction term allowed us to determine changes over time in socioeconomic inequalities in the study outcomes. For SHS exposure in children’s playgrounds, models did not include the survey year since no data were assessed for 2016. All analyses were weighted using child’s sex and age weights according to data from the National Statistics Institute of Spain to correct the study sample’s deviations from the Spanish population characteristics^20^. Analyses were conducted using STATA 15.1. A p<0.05 was considered statistically significant.

## RESULTS

As presented in Table 1, both samples (2016 and 2019) were similar in terms of children’s characteristics. There were some differences in the profile of the adult respondents, especially for the prevalence of smokers, which was higher in 2019 (22.0% vs 17.0%, p<0.001).

**Table 1:** Comparison of the children’s sociodemographic characteristics, adult respondents’ profile and the household socioeconomic position between 2016 and 2019, Spain

*Characteristics*	*2016 (N=2411) %*	*2019 (N=2412) %*	*p^a^*
**Children**			
**Sex**			
Girls	48.4	48.5	**0.987**
**Age** (years)			
0–3	30.4	29.5	0.449
4–7	34.3	33.5	
8–11	35.3	37.0	
**Country of birth**			
Spain	98.2	98.3	0.625
**Survey respondents**			
**Family relationship with the child**			
Father	36.3	28.2	**<0.001**
Mother	57.4	71.2	
Other	6.3	0.6	
**Age** (years)			
18–30	5.6	3.5	**<0.001**
31–40	40.4	41.7	
41–50	44.8	49.6	
51–60	6.4	4.5	
>60	2.9	0.7	
**Country of birth**			
Spain	87.5	89.4	**0.045**
**Smoking status**			
Smoker	17.0	22.0	**<0.001**
**Household SEP**			
**Education level of home main earner**			
Primary or lower	14.8	11.4	**<0.001**
Secondary	41.1	47.8	
University	44.1	40.8	

a Chi-squared test. SEP: socioeconomic position. Bold indicates p<0.05.

**Figure 1:**
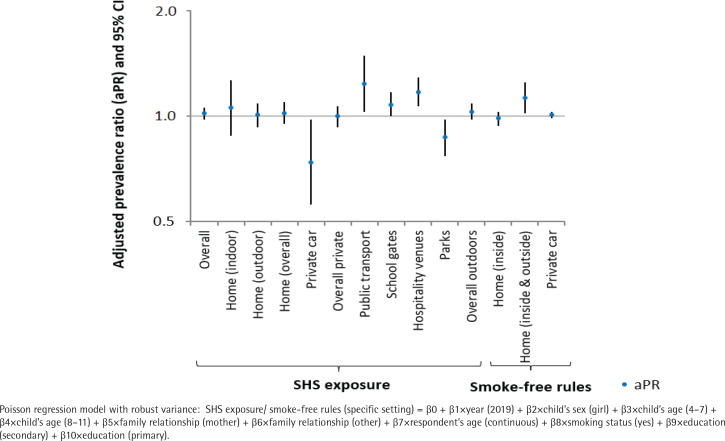
Adjusted prevalence ratios for children’s SHS exposure in private and outdoor public settings and the adoption of voluntary smoke-free rules between 2016 and 2019, Spain

Table 2 shows the prevalence of children exposed to SHS in Spain in different private and outdoor public settings and adoption of voluntary smoke-free rules in private settings, in 2016 and 2019. Figure 1 and Supplementary file Table S1 show adjusted prevalence ratios for SHS exposure and smoke-free rules between 2016 and 2019. Supplementary file Table S1 also includes crude analysis. The prevalence of children exposed to SHS in Spain was 70.5% in 2019. Almost 30% of children were exposed at home (9% indoors; 27.8% outdoors) in 2019. We did not find significant changes in overall exposure and exposure at home between 2016 and 2019. Exposure in private cars decreased in 2019 in comparison with 2016 (4.5% to 3.6%; APR=0.74; 95% CI: 0.56–0.98). Considering overall exposure in private settings (i.e. being exposed at home and/or car), 31.1% of children were exposed to SHS in 2019, and no significant changes were observed compared to 2016. Relative to 2016, models showed an increase of SHS exposure in 2019 in outdoor settings such as public transport stations (8.3% to 10.3%; APR=1.24; 95% CI: 1.03–1.49), and outdoor areas of hospitality venues (25.9% to 31%; APR=1.17; 95% CI: 1.07–1.29). SHS exposure at parks decreased in 2019 with respect to 2016 (20.6% to 17.7%; APR=0.87; 95% CI: 0.77–0.98). Changes in the prevalence of exposure at school entrances were not statistically significant (31.6% to 35.1%; APR=1.08; 95% CI: 1.00–1.17). The overall exposure in outdoor public settings (i.e. being exposed in at least one of the outdoor public settings studied) was 59.2% in 2019, with no significant changes observed between survey years. Moreover, 15% of children were exposed to SHS in children’s playgrounds in 2019. A significant increase in the adoption of smoke-free homes (banned inside and outside) was observed between 2016 and 2019 (23.2% to 25.1%; APR=1.13; 95% CI: 1.02–1.25).

**Table 2:** Prevalence of SHS exposure in private and outdoor public settings in children aged <12 years, and adoption of voluntary smoke-free rules in private settings, in 2016 and 2019, Spain

	*2016 (N= 2411)*		*2019 (N=2412)*	
	*Unadjusted*		*Unadjusted*	
	*%*	*95% CI*	*%*	*95% CI*
**Overall SHS exposure**				
Overall	67.1	65.1–69.0	70.5	68.6–72.4
**SHS exposure in private settings**				
Home (indoors)	7.6	6.5–8.6	9.0	7.8–10.1
Home (outdoors)	24.4	22.7–26.2	27.8	26.0–29.6
Home (overall)	25.7	24.0–27.5	29.8	28.0–31.7
Car	4.5	3.7–5.4	3.6	2.9–4.3
Overall private settings	27.7	25.9–29.5	31.1	29.2–33.0
**SHS exposure in outdoor settings**				
Public transport stations	8.3	7.1–9.4	10.3	9.1–11.6
School and nursery gates	31.6	29.8–33.5	35.1	33.1–37.1
Outdoor areas of hospitality venues	25.9	24.1–27.7	31.0	29.1–32.9
Parks	20.6	18.9–22.3	17.7	16.2–19.3
Children’s playgrounds	NA	NA	15.0	13.5–16.4
Overall outdoor settings	56.4	54.4–58.4	59.2	57.0–61.3
**Adoption of smoke-free rules**				
**In homes**				
Only inside	61.3	59.4–63.3	60.3	58.3–62.3
Inside and outside	23.2	21.5–24.9	25.1	23.4–26.9
**In private cars**				
Banned	91.0	89.9–92.2	91.5	90.4–92.6

NA: not assessed. Percentages were calculated with margins when the other independent variables have their values at their means.

Significant differences were observed among SHS exposure according to household’s SEP (Table 3). Higher overall SHS exposure was observed among children living in households whose main earner had primary or lower education level compared to those with university studies, in both survey years (APR=1.09; 95% CI: 1.01–1.18). A higher prevalence of overall SHS exposure was also found among households whose main earner had secondary studies compared to university education in 2016 (APR=1.10; 95% CI: 1.04–1.17). Higher SHS exposure at home (overall, indoors, and outdoors) was observed in 2016 and 2019 among children living in households whose main earner had primary or secondary studies in comparison with those with university education. In 2019, we found lower SHS exposure in outdoor hospitality venues among households whose main earner had primary (APR=0.76; 95% CI: 0.61–0.94) or secondary education (APR=0.82; 95% CI: 0.72–0.93) compared to those with university studies. No interaction was found between year and education level for any setting, suggesting changes in children’s SHS exposure between survey years were similar for all levels of education.

**Table 3:** Adjusted prevalence ratio of SHS exposure among children aged <12 years by education level of the home main earner (household SEP), 2016 and 2019, Spain

	*2016 (N=2411)*		*2019 (N=2412)*		*p ^b^*
	*APR ^a^*	*95% CI*	*APR ^a^*	*95 % CI*	
**Overall exposure**					
University ®	1		1		
Secondary	**1.10**	**1.04–1.17**	1.03	0.97–1.09	0.116
Primary or lower	**1.09**	**1.01–1.18**	**1.09**	**1.01–1.18**	1.000
**Private settings**					
**Home** (indoors)					
University ®	1		1		
Secondary	**1.46**	**1.05–2.03**	**1.51**	**1.13–2.03**	0.878
Primary or lower	**2.15**	**1.48–3.11**	**1.90**	**1.31–2.77**	0.646
**Home** (outdoors)					
University ®	1		1		
Secondary	**1.20**	**1.05–1.36**	1.09	0.97–1.22	0.276
Primary or lower	**1.33**	**1.14–1.55**	**1.26**	**1.07–1.49**	0.644
**Home** (overall)					
University ®	1		1		
Secondary	**1.19**	**1.06–1.34**	**1.12**	**1.00–1.25**	0.446
Primary or lower	**1.28**	**1.11–1.48**	**1.30**	**1.11–1.51**	0.925
**Car**					
University ®	1		1		
Secondary	1.40	0.92–2.15	0.99	0.63–1.57	0.272
Primary or lower	1.62	0.97–2.70	1.06	0.55–2.06	0.320
**Overall private settings**					
University ®	1		1		
Secondary	**1.19**	**1.06–1.34**	**1.14**	**1.02–1.27**	0.545
Primary or lower	**1.28**	**1.11–1.47**	**1.37**	**1.18–1.60**	0.476
**Outdoor settings**					
**Public transport stations**					
University ®	1		1		
Secondary	0.94	0.70–1.27	1.11	0.85–1.45	0.419
Primary or lower	1.17	0.79–1.73	1.08	0.72–1.60	0.763
**Schools and nursery gates**					
University ®	1		1		
Secondary	**1.21**	**1.07–1.38**	1.09	0.97–1.23	0.213
Primary or lower	1.04	0.86–1.25	1.05	0.87–1.26	0.937
**Outdoor areas of hospitality venues**					
University ®	1		1		
Secondary	0.87	0.75–1.01	**0.82**	**0.72–0.93**	0.518
Primary or lower	0.93	0.76–1.14	**0.76**	**0.61–0.94**	0.164
**Parks**					
University ®	1		1		
Secondary	1.18	0.99–1.40	0.96	0.79–1.16	0.122
Primary or lower	1.07	0.84–1.38	1.05	0.79–1.39	0.900
**Children’s playgrounds**					
University ®	NA		1		
Secondary	NA	NA	1.04	0.84–1.28	NA
Primary or lower	NA	NA	1.05	0.77–1.44	NA
**Overall outdoor settings**					
University ®	1		1		
Secondary	1.07	0.99–1.16	1.01	0.94–1.09	0.322
Primary or lower	1.02	0.92–1.14	1.03	0.91–1.15	0.959

a APR: adjusted prevalence ratio. NA: not assessed.

b p-value for the interaction term between year and education level of home main earner.

In bold associations with p<0.05. Poisson regression model with robust variance: SHS exposure (specific setting) = β0 + β1×year (2019) + β2×education (secondary) + β2×education (primary) + β3×year (2019)×education (secondary) + β3×year (2019)×education (primary) + β4×child’s sex (girl) + β5×child’s age (4–7) + β6×child’s age (8–11) + β7×family relationship (mother) + β8×family relationship (other) + β9×respondent’s age (continuous) + β8×smoking status (yes). Percentages were calculated with margins when the other independent variables have their values at their means. ® Reference categories.

Regarding smoke-free rules, we also found some differences according to household’s SEP (Table 4). For both survey years, we observed a lower percentage of households adopting indoor home smoke-free rules among those whose main earner had primary or lower studies (2016, APR=0.89; 95% CI: 0.80–0.98; 2019, APR=0.88; 95% CI: 0.79–0.99) or secondary education (2016, APR=0.93; 95% CI: 0.87–0.99; 2019, APR=0.95; 95% CI: 0.89–1.02) compared to those with university studies. Yet, no differences according to household SEP were found in the prevalence of households with smoke-free rules that included both indoor and outdoor areas. No interaction was found between year and education level, suggesting socioeconomic inequalities in home smoke-free rules remained similar over time. We also found a lower prevalence of smoke-free rules in cars among households whose main earner had secondary (APR=0.96; 95% CI: 0.94–0.99), primary, or lower education level (APR=0.94; 95% CI: 0.90–0.98) with respect to those with university studies in 2016. Interaction between education level and year was found for secondary studies, suggesting a higher increase in the adoption of smoke-free rules among these group compared with households whose main earner had university studies.

**Table 4:** Adjusted prevalence ratio of families with children aged <12 years adopting smoke-free rules in private settings, by education level of the home main earner (household SEP), 2016 and 2019, Spain

	*2016 (N=2411)*		*2019 (N=2412)*		
	*APR ^a^*	*95% CI*	*APR ^a^*	*95% CI*	*p ^b^*
**Smoke-free rules in homes**					
**Only inside**					
University ®	1		1		
Secondary	**0.93**	**0.87–0.99**	0.95	0.89–1.02	0.637
Primary or lower	**0.89**	**0.80–0.98**	**0.88**	**0.79–0.99**	0.963
**Inside and outside**					
University ®	1		1		
Secondary	1.13	0.97–1.32	0.95	0.82–1.10	0.114
Primary or lower	1.13	0.90–1.42	0.93	0.73–1.19	0.253
**Smoke-free rules in private cars**					
University ®	1		1		
Secondary	**0.96**	**0.94–0.99**	1.00	0.97–1.02	**0.039**
Primary or lower	**0.94**	**0.90–0.98**	0.99	0.95–1.04	0.106

a APR: adjusted prevalence ratio.

b p-value for the interaction term year×education level of home main earner.

In bold associations with p<0.05. Poisson regression model with robust variance: Smoke-free rules (specific setting) = β0 + β1×year (2019) + β2×education (secondary) + β2×education (primary) + β3×year (2019)×education (secondary) + β3×year (2019)×education (primary) + β4×child’s sex (girl) + β5×child’s age (4–7) + β6×child’s age (8–11) + β7×family relationship (mother) + β8×family relationship (other) + β9×respondent’s age (continuous) + β8×smoking status (yes). Percentages were calculated with margins when the other independent variables have their values at their means. ® Reference categories.

## DISCUSSION

This study shows a high prevalence of SHS exposure among children aged <12 years in Spain, both in private and public settings, in 2016 and 2019. Nearly three out of four children were found to be exposed to SHS; about one-third in private settings and almost two out of three in outdoor public venues. The exposure increased in 2019 at public transport stations and outdoor hospitality venues and decreased in cars and parks. Results also show an increase in the prevalence of household’s becoming smoke-free. Our findings suggest socioeconomic inequalities in SHS exposure and the adoption of smoke-free rules persisted in 2019 in homes but decreased in cars.

According to our data, in 2019, 9% of the children were exposed to SHS inside the home and about one out of four children at the household’s outdoor areas, with a similar prevalence for 2016. Smoking in balconies or terraces is frequently used as a preventive measure to avoid indoor SHS exposures. Yet, the health risk of smoking in these outdoor spaces might be underestimated^21,22^ since SHS can easily diffuse from outdoor to indoor areas^21,23^. Our results confirm the need to keep raising public awareness of health risks from home smoking, especially in the adjacent outdoor areas. Regarding private cars, we found SHS exposure decreased between 2016 and 2019, suggesting a potential increase in parental awareness of the harmful effect of smoking inside small, enclosed spaces, where the exposure can be very intense^24^. Even so, children are still exposed to SHS when travelling by car. As in other countries such as the United Kingdom or Italy, smoking inside cars should be banned in Spain, especially when minors are present.

Over one-third of children aged <12 years in Spain were exposed to SHS in school entrances which remained similar between the two years of this study. These results are aligned with prior research confirming smoking near Spanish schools^25,26^. Unexpectedly, we found an increase in SHS exposure at public transport stations and outdoor hospitality venues. A greater awareness of the health effects from SHS exposures over time might explain the present results. Parents with higher awareness might be more likely to report someone has smoked near their child. Our results support the need for an extension of smoking bans to cover the school entrances, and for total smoking bans in all outdoor hospitality areas to protect children’s health. People generally believe the health risks of outdoor SHS exposure are low. Yet, outdoor SHS can reach indoor levels during periods of active smoking^27^. Furthermore, there is no safe level of exposure to SHS^1^ and even brief exposures could be detrimental to health^28^. Moreover, visibility of smoking could lead children to normalize tobacco use and become more susceptible to initiating smoking during adolescence or adulthood^7^. Notably, the adoption of smoke-free policies might help to encourage people to adopt smoke-free homes and cars^29^.

The decrease in SHS exposure in parks between survey years is likely due to a potential overestimation of the reported exposure in 2016. Since we did not collected data on SHS exposure in playgrounds in 2016, respondents may have included the exposure in this setting within their responses to the park question, in contrast to 2019. Our results also indicate non-compliance and a need for greater enforcement of smoking bans in children’s playgrounds in Spain. Smoking at these venues is banned since 2011 in Spain^8^, still 15% of the children were exposed to SHS in playgrounds in 2019 according to our data. In this line, other studies from 2017–2018 reported presence of people smoking^25^ and detectable levels of nicotine^30^ in these child-related outdoor areas in Spain.

Our findings reveal nearly 2 out of 3 families living with children had adopted smoke-free rules inside the house in 2016 and 2019. Only nearly one out of four reported doing so at both household’s indoor and outdoor areas in 2016, with a slightly increase in 2019. Despite that, we did not observe a decline in SHS exposure at household outdoors. High levels of nicotine concentrations inside the house have been associated with less restrictive household smoking bans. Detectable levels of nicotine have even been observed when someone had smoked only outside the house^21^. This highlights the importance of adopting both indoor and outdoor smoke-free rules to fully prevent children from SHS exposures at home.

According to our data, children in households whose main earner had a lower education level were more exposed to SHS in 2016, this pattern persisting in 2019. This is consistent with previous studies showing socioeconomic inequalities in SHS exposure at home persists over the years^10,11^. Social inequalities in parental smoking could partly explain these patterns, since children whose parents smoke are more likely to be exposed to SHS^31^. On the other hand, prior research in Montreal (Canada) suggests social inequalities in children’s SHS exposure in private cars, being those children belonging to the most disadvantaged areas more likely to be exposed to SHS when travelling in cars^32^. Yet, no significant differences were found across the education groups in our study.

In contrast to 2016, we observed socioeconomic inequalities in SHS exposure at outdoor hospitality venues in 2019, being the exposure higher among households whose main earner had a higher education level. Our interaction analysis, however, did not reveal a significant increase in inequalities between survey years. The observed differences in the reported exposure according to education level in 2019 may be attributed to an enhanced awareness of SHS risks particularly among households with higher education level, rather than a real increase in exposure. This hypothesis aligns with a recent study that found no significant disparities in objective SHS exposure^33^.

Our data, similar to other studies^10,34^, showed that indoor smoke-free rules at home were less common among households whose main earner had a lower education level, this pattern persisting throughout the survey years and concurring with the socioeconomic inequalities observed in SHS exposure in homes. Less educated parents more likely underestimate the risks of SHS^31^, and are thus less likely to adopt voluntary smoke-free rules. We did not find significant differences in the adoption of rules at home by household SEP when considering those families who banned both the indoor and outdoor areas. According to a previous study, in Wales, children from poorer families were less likely to report smoke-free rules in cars^35^. We observed inequalities in the adoption of car smoke-free rules according to household SEP in 2016 but not in 2019, suggesting a decrease of such inequalities.

### Limitations

Some limitations to this research should be considered. First, the use of a questionnaire to estimate SHS exposure could lead to potential recall and social desirability biases. However, the questionnaire was designed based on a previous questionnaire used to estimate adult’s exposure to SHS^15,16^, and that was adapted for children. Also, it was piloted and questions about home exposure were validated using environmental nicotine concentrations as a gold standard^36^. To reduce recall bias, we asked for exposures during the previous week. Another potential limitation is the use of a repeat cross-sectional design rather than a longitudinal design. However, the limited number of homes re-interviewed in 2019 did not allow us to follow a longitudinal design. Thus, data were weighted to ensure the sample was representative of children’s Spanish population regarding child’s age and sex in 2016 and 2019^20^. Our findings might be limited by residual confounding. However, our analysis considered various potential confounding factors such as sociodemographic characteristics of children (sex and age), respondents (relationship with the child, age, country of birth, and smoking status), and household SEP (education level of home main earner). Finally, our results have limited generalizability to other countries.

This study has however been an opportunity to evaluate changes in the prevalence of SHS exposure in multiple settings and in the adoption of smokefree homes and cars among children, a vulnerable and sensitive population to SHS. Importantly, SHS exposure was evaluated in a nationally representative sample of Spanish children younger than 12 years. Moreover, this study provides information on recent changes in children’s socioeconomic inequalities in SHS exposure and smoke-free rules.

## CONCLUSIONS

Our data show SHS exposure continues to be a major health hazard for children, with high prevalences of exposure in several private and public settings both in 2016 and 2019. We observed the reported exposure increased in 2019 at public transport stations and outdoor hospitality venues. Moreover, SHS exposure was reported in child-related venues where smoking is already banned in Spain, such as children’s playgrounds. More enforcement and compliance as well as expanding the number of smoke-free settings covered in the current Spanish legislation should be considered to protect children from the harmful effects of tobacco and denormalize smoking behaviour^37^. Also, according to our findings, socioeconomic inequalities in SHS exposure have persisted between 2016 and 2019 at home, where children spend most of their time. Thus, evidence-based approaches targeting families with a disadvantaged SEP should be adapted and implemented to promote smoke-free homes^38^.

## Supplementary Material



## Data Availability

The data supporting this research are available from the authors on reasonable request.
