# Supplementary material for: Changes in prevalence and socioeconomic inequalities in secondhand smoke exposure in Spanish children, 2016–2019
Source: Tob Induc Dis. 2024 Jun 21;22:10.18332/tid/189392. doi: 10.18332/tid/189392 (PMC11191057; doi:10.18332/tid/189392)
Supplement: Supplementary file 1 [file TID-22-116-s1.pdf]

Supplementary material

Table S1. Crude and adjusted prevalence ratios of SHS exposure in private and outdoor public settings in children younger than 12 years old, and adoption of voluntary smoke-free rules in private settings, in 2016 and 2019, Spain.

	Change 2016-2019			
	Crude		Adjusted	
	cPR[a]	95% CI	aPR[b]	95% CI
**Overall SHS exposure**				
Overall	**1.05**	**1.01 - 1.09**	1.02	0.98 - 1.06
**SHS exposure in private settings**				
Home (indoors)	1.19	0.98 - 1.43	1.06	0.88 - 1.27
Home (outdoors)	**1.14**	**1.03 - 1.25**	1.01	0.93 - 1.09
Home (overall)	**1.16**	**1.06 - 1.27**	1.02	0.95 - 1.10
Car	0.79	0.60 - 1.05	**0.74**	**0.56 - 0.98**
Overall private settings	**1.12**	**1.03 - 1.22**	1.00	0.93 - 1.07
**SHS exposure in outdoor settings**				
Public transport stations	**1.25**	**1.04 - 1.50**	**1.24**	**1.03 - 1.49**
School & nursery gates	**1.11**	**1.02 - 1.20**	1.08	1.00 - 1.17
Outdoor areas of hospitality venues	**1.20**	**1.09 - 1.31**	**1.17**	**1.07 - 1.29**
Parks	**0.86**	**0.76 - 0.97**	**0.87**	**0.77 - 0.98**
Children's playgrounds	na	na	na	na
Overall outdoor settings	1.05	1.00 - 1.10	1.03	0.98 - 1.09
**Adoption of smoke-free rules**				
**In homes**				
Only inside	0.98	0.94 - 1.03	0.99	0.94 - 1.03
Inside and outside	1.08	0.98 - 1.20	**1.13**	**1.02 - 1.25**
**In private cars**				
Banned	1.00	0.99 - 1.02	1.01	0.99 - 1.03

CI: Confidence Interval; na: Not assessed.; In bold associations with a p-value < 0.05.

[a] Crude Prevalence Ratio (cPR). Crude Poisson regression model with robust variance: SHS exposure/ Smoke-free rules (specific setting) = $\beta0 + \beta1 \cdot$year(2019)

[b] Adjusted Prevalence Ratio (aPR). Adjusted Poisson regression model with robust variance: SHS exposure/ Smoke-free rules (specific setting) = $\beta0 + \beta1 \cdot$year(2019) + $\beta2 \cdot$child's sex(girl) + $\beta3 \cdot$child's age(4-7) + $\beta4 \cdot$child's age(8-11) + $\beta5 \cdot$family relationship(mother) + $\beta6 \cdot$family relationship(other) + $\beta7 \cdot$respondent's age(continuous) + $\beta8 \cdot$smoking status(yes) + $\beta9 \cdot$education(secondary) + $\beta10 \cdot$education(primary)

© 2024 Sanz-Mas M. et al.
